# Gentamicin combination treatment is associated with lower mortality in patients with invasive listeriosis: a retrospective analysis

**DOI:** 10.1007/s15010-024-02330-w

**Published:** 2024-07-04

**Authors:** Jan P. Sutter, Lorenz Kocheise, Jan Kempski, Martin Christner, Dominic Wichmann, Hans Pinnschmidt, Stefan Schmiedel, Ansgar W. Lohse, Samuel Huber, Thomas Theo Brehm

**Affiliations:** 1 I. Department of Medicine, University Medical Center Hamburg-Eppendorf, Martinistraße 52, 20246 Hamburg, Germany; 2 German Center for Infection Research (DZIF), Partner-Site Hamburg-Lübeck-Borstel-Riems, 20246 Hamburg, Germany; 3 Division of Clinical Infectious Diseases, Research Center Borstel, 23845 Borstel, Germany; 4 Institute of Microbiology, Virology and Hygiene, University Medical Center Hamburg-Eppendorf, 20246 Hamburg, Germany; 5 Department of Intensive Care Medicine, University Medical Center Hamburg-Eppendorf, 20246 Hamburg, Germany; 6 Center for Experimental Medicine, Institute for Medical Biometry & Epidemiology, University Medical Center Hamburg-Eppendorf, 20246 Hamburg, Germany

**Keywords:** Listeriosis, Gentamicin, Neurolisteriosis, Combination treatment

## Abstract

**Purpose:**

*Listeria monocytogenes* causes severe bacterial infections with the highest mortality rate among foodborne pathogens in Europe. Combination treatment with ampicillin and gentamicin is recommended for invasive manifestations. However, evidence to support this treatment approach remains limited due to a lack of randomised controlled trials. To explore this critical issue further, we conducted this retrospective, single-center study.

**Methods:**

We identified all patients hospitalized with invasive listeriosis at the University Medical Center Hamburg-Eppendorf between 2009 and 2020 and analyzed the effect of gentamicin combination treatment versus monotherapy on 90-day mortality.

**Results:**

In total, 36 patients with invasive listeriosis were included, of which 21 patients received gentamicin combination treatment and 15 received monotherapy. The mean age-adjusted Charlson Comorbidity Index (aaCCI) value was lower in the gentamicin combination treatment group (5.4 vs. 7.4). Neurolisteriosis was more common in the gentamicin group (81% vs. 20%). The 90-day mortality was with significantly lower in the gentamicin combination treatment group (10%) compared to the monotherapy group (60%). Multivariable cox regression analysis, adjusted for a propensity score computed based on neurolisteriosis, aaCCI and sex, revealed a significantly reduced hazard ratio of 0.07 (95% CI: 0.01–0.53, *p* = 0.01) for 90-day mortality for the gentamicin combination treatment.

**Conclusion:**

This retrospective study highlights the benefit of gentamicin combination treatment in reducing the 90-day mortality rate among patients with invasive listeriosis. The high prevalence of monotherapy in this study cohort raises concerns about the adequacy of antibiotic therapy in clinical practice.

## Introduction

*Listeria monocytogenes*, the causative pathogen of listeriosis is associated with severe bacterial foodborne infections and represents a significant public health threat [1]. Non-perinatal cases are associated with a mortality rate of approximately 26%, which is the highest rate of any foodborne pathogens in Europe [2]. The incidence of laboratory-confirmed cases of listeriosis in Europe ranges from 0.3 to 0.6 cases per 100,000 persons [2]. Listeriosis can present as three invasive forms: bacteraemia, neurolisteriosis, and maternal-neonatal infection. Epidemiological studies have identified several risk factors for bacteraemia and neurolisteriosis, including advanced age, immunosuppression, malignancy, liver cirrhosis, diabetes mellitus, and alcoholism [3]. Unfortunately, the mortality from invasive listeriosis has not improved in recent decades [2]. The rarity and dispersion of listeriosis cases has made it difficult to conduct clinical trials, resulting in a lack of robust evidence to guide treatment. The most common treatment for invasive listeriosis is the combination of ampicillin and gentamicin [4]. However, due to the absence of randomised controlled trials, evidence supporting this treatment approach remains limited. While some studies have questioned the efficacy of gentamicin combination therapy [5], others have shown a survival advantage compared with beta-lactam monotherapy [2]. We conducted a retrospective single-center study to investigate the possible association between the use of additional gentamicin and improved survival rates in a real-world cohort of patients with invasive listeriosis hospitalized at the University Medical Center Hamburg-Eppendorf.

## Methods

### Selection of the study cohort

We identified all patients hospitalized with invasive listeriosis at the University Medical Center Hamburg-Eppendorf between 2009 and 2020. Comprehensive data on demographic information, past medical history, antibiotic treatment and disease course were systematically collected from electronic patient charts. To assess the impact of added gentamicin on 90-day mortality, we included all patients with *L. monocytogenes* bacteraemia and patients with neurolisteriosis who received adequate antibiotic therapy. Adequate antibiotic therapy was defined as treatment with ampicillin, trimethoprim/sulfamethoxazole, or meropenem with or without gentamicin.

Bacteraemia was defined by the isolation of *L. monocytogenes* from blood cultures, excluding cases with neurolisteriosis. Neurolisteriosis was defined as isolation of *L. monocytogenes* from cerebrospinal fluid (CSF) by culture or polymerase chain reaction (PCR) or by a combination of positive imaging (cranial computed tomography or magnetic resonance imaging), bacteraemia and compatible symptoms. An a priori decision was made to exclude patients with maternal-neonatal infection or other manifestations from the analysis. Maternal-neonatal infection was defined as isolation of *L. monocytogenes* from pregnant women, placental tissue or infants aged three months or younger. Comorbidities were quantified using the age-adjusted Charlson Comorbidity Index (aaCCI) [6]. As previous studies suggest that the use of proton pump inhibitors (PPIs) may increase the risk of developing an invasive *L. monocytogenes* infection [7], we assessed whether PPIs were included in the medication regimen at the time of admission for all patients. Serum creatinine levels were assessed upon admission for all patients, as acute or chronic renal disease may prompt physicians to decide against gentamicin administration due to concerns about nephrotoxic effects.

### Statistical analyses

Two-sided tests were performed using Fischer’s exact test. Continuous variables were analyzed using the Mann-Whitney test. The survival times of the gentamicin combination treatment group and the monotherapy group were analyzed using the log-rank test and plotted on Kaplan-Meier curves. An a priori decision was made to consider neurolisteriosis, sex, aaCCI and treatment group as independent variables affecting mortality from *L. monocytogenes*, as suggested by results of previous studies [2]. To compute a propensity score representing the probability of assigning the gentamicin combination treatment to a given patient, the variables neurolisteriosis, sex and aaCCI were forced into a logistic regression equation, using treatment group as dependent variable. Multivariable Cox regression analyses were then performed to assess the risk factors for 90-day mortality. In analysis 1, neurolisteriosis, sex, aaCCI and treatment group were forced into the regression equation while in analysis 2, only treatment group and the propensity score were forced into the equation. To ensure that multicollinearity amongst independent variables was not of concern, the variance inflation factors were computed and evaluated for all independent variables in the Cox regression equations. For all statistical analyses p-values below 0.05 were considered significant. Statistical analyses were performed using GraphPad Prism (version 10.1.2) and IBM SPSS Statistics (version 29.0.1.0).

## Results

### Study population

**Fig. 1:**
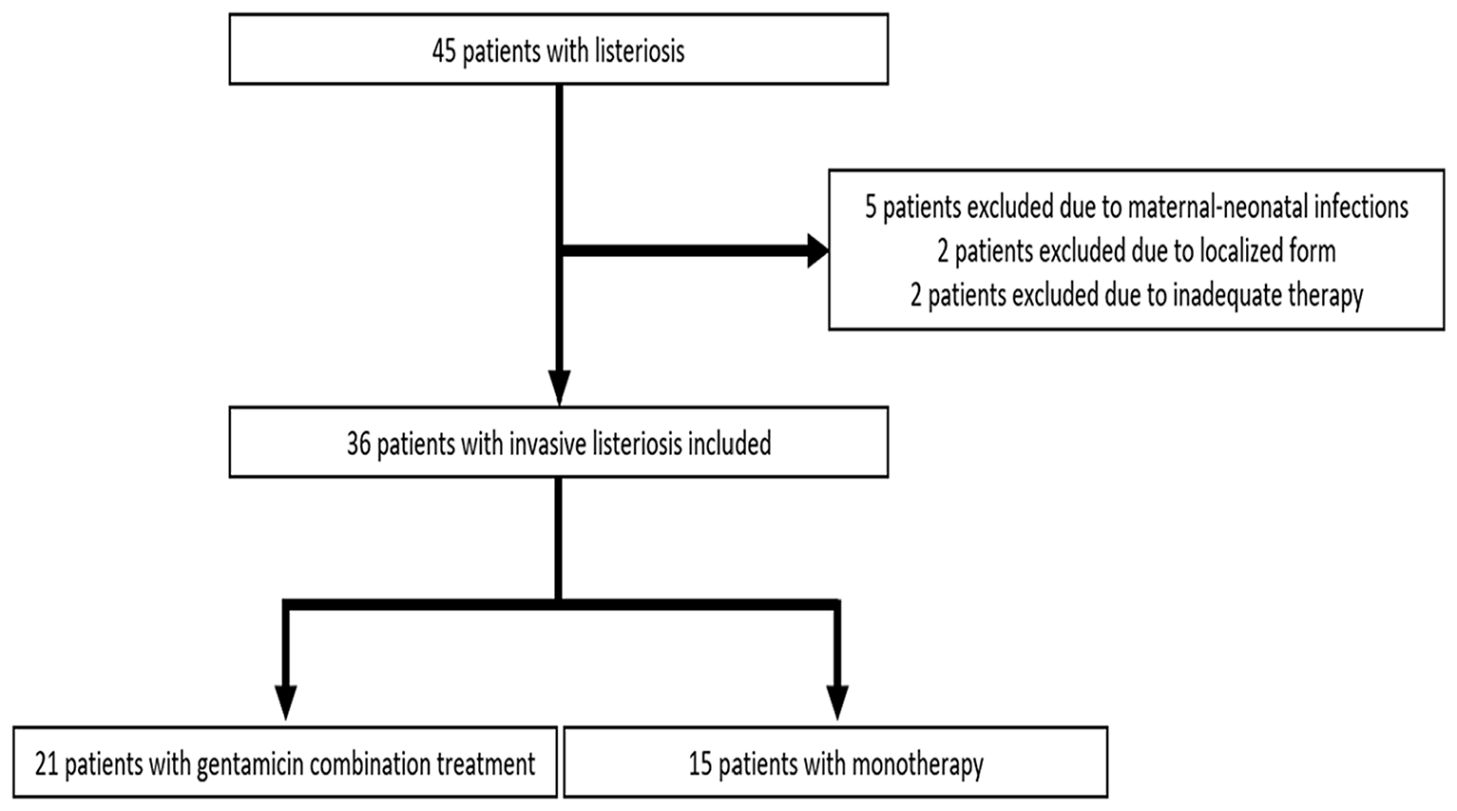
Patient selection

Between 2009 and 2020, a total of 45 patients with listeriosis were treated at our center (Fig. 1). Two patients were excluded because of maternal infection and three because of neonatal infection. Two patients were excluded because they had isolated peritonitis with detection of *L. monocytogenes* in ascites but no bacteraemia or neurolisteriosis. Two patients were excluded from the analysis because they did not receive adequate antibiotic treatment as defined by our inclusion criteria. The final analysis included 36 patients with invasive listeriosis, of whom 21 (58%) received gentamicin combination treatment and 15 (42%) received monotherapy. Patients in the gentamicin combination treatment group (median age 60 years [IQR 52–71]) were significantly younger than those in the monotherapy group (median age 73 years [IQR 67–78], *p* = 0.012) (Table 1). There were no differences in male-to-female ratio between the groups. The mean aaCCI was significantly lower in the gentamicin combination treatment than in the monotherapy group (5.4 vs. 7.4, *p* = 0.023). The most common comorbidities were cardiovascular disease, chronic liver disease, diabetes mellitus, kidney disease and neoplasia. The majority of patients in the gentamicin combination treatment group (90%, *n* = 19) and in the monotherapy group (100%, *n* = 15), were immunocompromised. Steroid use was significantly higher in the gentamicin combination treatment group (86%, *n* = 18) compared to the monotherapy group (40%, *n* = 6, *p* = 0.01). In both groups, more than 80% of the patients were taking PPIs as part of their long-term medication. In the gentamicin combination treatment group, 17 patients (81%) had neurolisteriosis, a significantly higher proportion compared to the monotherapy group (20%, *n* = 3; *p* < 0.001).

**Table 1:** Overview of patient characteristics and disease course

	Gentamicin combination treatment group (*n* = 21)	Monotherapy group (*n* = 15)	*p* value
Age (years), median (IQR)	60 (52–71)	73 (67–78)	0.01
Male, n (%)	15 (65)	8 (53)	0.31
Immunosuppression, n (%)	19 (90)	15 (100)	1.0
aaCCI, mean (SD)	5.4 (2.9)	7.4 (2.1)	0.02
Chronic kidney disease, n (%)	2 (10)	4 (27)	0.21
Proton pump inhibitor use	17 (81)	14 (93)	0.38
Time from symptom onset to diagnosis (days), median (IQR)	3 (2–5)^a^	3 (2–4)^b^	0.84
Neurolisteriosis, n (%)	17 (81)	3 (20)	< 0.001
Fever (> 38 °C), n (%)	21 (100)	9 (60)	0.003
Admission to intensive care unit, n (%)	14 (67)	5 (33)	0.09
90-day mortality, n (%)	2 (10)	9 (60)	0.002

Legend: Immunosuppressive comorbidities included: daily alcohol consumption of more than three drinks per day, cirrhosis, diabetes mellitus, end-stage renal disease, cancer of a solid organ, haematological malignancy, haemopoietic stem cell transplantation, solid organ transplantation, asplenia, pre-existing neutropenia, pre-existing lymphopenia, HIV infection, inflammatory bowel disease, inflammatory rheumatic disease, over 70 years of age, prescription of corticosteroids or other immunosuppressants. Interquartile range (IQR), number (n), standard deviation (SD). ^a^data available for *n* = 18 patients, ^b^data available for *n* = 11 patients

The mean time from symptom onset to diagnosis was three days in both groups. We were able to collect this data in 18 of 21 patients in the gentamicin combination group and in 11 of 15 patients in the monotherapy group. All patients in the gentamicin combination treatment group, but only 60% (*n* = 9) patients in the monotherapy group presented with febrile illness (*p* = 0.003). No significant difference was found in the initial serum creatinine between both groups.

### Antibiotic treatment

The median duration of antibiotic therapy was 21 days (IQR: gentamicin combination treatment 21–28, monotherapy 12–21) in both groups. Overall, ampicillin was the most commonly used antibiotic in both groups. In the gentamicin combination treatment group, 20 patients (95%) received ampicillin, seven (33%) received meropenem, and two (10%) received trimethoprim/sulfamethoxazole. Of the patients in the monotherapy group, eleven (73%) were treated with ampicillin, six (40%) with meropenem, and three (20%) with trimethoprim/sulfamethoxazole (Table 2). All patients in the gentamicin combination treatment group received gentamicin for at least four days with an average duration of 11 days. Overall, 67% (*n* = 14) of patients in the gentamicin combination treatment group and 33% (*n* = 5) of patients in the monotherapy group were admitted to the intensive care unit (ICU) (Table 1). Of all patients admitted to the ICU, 68% (*n* = 13) had neurolisteriosis and 32% (*n* = 6) had bacteraemia. Logistic regression results revealed that the presence of neurolisteriosis increases the likelihood of patients being assigned to gentamicin combination treatment. While sex and aaCCI were not significantly associated with this treatment, the respective odds ratios suggest a higher likelihood for male patients to receive gentamicin and a lower likelihood for patients with high aaCCI values (Table 3).

**Table 2:** Antibiotic treatment regimen. Number (n)

	Gentamicin combination treatment group (*n* = 21)	Monotherapy group (*n* = 15)	*p* value
Ampicillin, n (%)	20 (95)	11 (73)	0.14
Trimethoprim/sulfamethoxazole, n (%)	2 (10)	3 (20)	0.63
Meropenem, n (%)	7 (33)	6 (40)	0.74

**Table 3:** Results of a logistic regression to compute propensity scores, representing the probability of gentamicin treatment = 1, based on aaCCI, presence of neurolisteriosis and male sex. Odds ratio (OR), confidence interval (CI), age-adjusted Charlson Comorbidity Index (aaCCI)

	OR	95%CI for OR	*p* value	Nagelkerke’s *R*^2^
aaCCI	0.74	0.49–1.12	0.15	
Neurolisteriosis	35.57	3.21-393.64	0.004	0.58
Male sex	6.26	0.59–66.74	0.129	

### Clinical outcomes

**Fig. 2:**
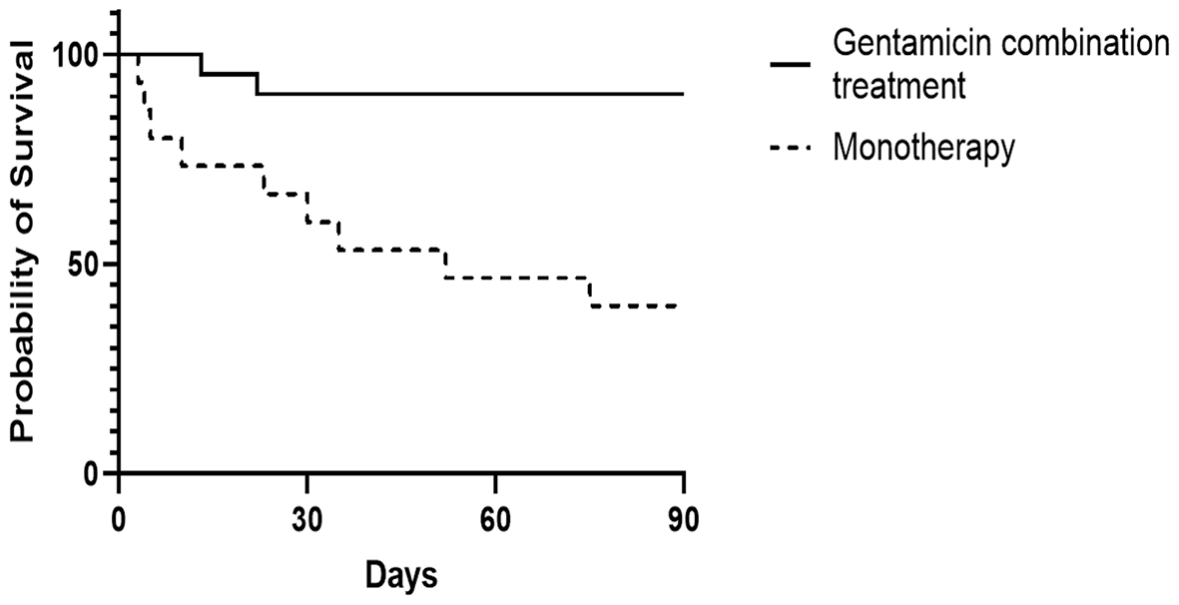
Kaplan-Meier Survival Analysis: Impact of gentamicin combination treatment and monotherapy on 90-day mortality (Log-rank Test, HR = 0.12, 95% CI: 0.3–0.41, *p* = 0.001)

All patients who survived had an outpatient follow-up visit to perform a 90-day mortality analysis. A significant difference in 90-day mortality was observed between the two subgroups. Among patients receiving gentamicin combination treatment, two patients (10%) died within 90 days, compared to nine patients (60%) in the monotherapy group (*p* = 0.002) (Table 1). Kaplan-Meier survival analysis indicated a significant difference in survival between the gentamicin combination treatment and monotherapy groups (hazard ratio [HR] = 0.12, 95% CI: 0.3–0.41, *p* = 0.001) (Fig. 2). In a multivariable Cox regression analysis, the HR for 90-day-mortality for gentamicin combination treatment was 0.06 (95% CI: 0.01–0.52, *p* = 0.01), indicating a significantly lower 90-day mortality risk compared with the monotherapy group, while the effects of aaCCI, sex, and neurolisteriosis were not significant (Table 4, analysis 1). When the three latter variables were replaced by the propensity score in the Cox regression, the effect of the treatment group remained almost the same, as indicated by the HR (HR = 0.07, 95% CI: 0.01–0.53, *p* = 0.01; Table 4, analysis 2).

**Table 4:** Multivariable Cox Regression Analysis of Mortality Factors: analysis 1: impact of gentamicin, neurolisteriosis, sex, and aaCCI on 90-day mortality. Analysis 2: impact of gentamicin and propensity score on 90-day mortality. Hazard ratio (HR), confidence interval (CI), age-adjusted Charlson Comorbidity Index (aaCCI)

Analysis		HR	95%CI for HR	*p*-value
1	Gentamicin combination treatment	0.06	0.01–0.52	0.01
	aaCCI	1.27	0.98–1.64	0.07
	Male sex	1.39	0.282–6.98	0.69
	Neurolisteriosis	3.28	0.48–22.48	0.23
2	Gentamicin combination treatment	0.06	0.01–0.52	0.01
	Propensity score	2.97	0.19–46.14	0.437

## Discussion

In this retrospective analysis, we aimed to determine the effect of gentamicin combination treatment on the clinical outcome of patients with invasive listeriosis who were hospitalised at the University Medical Center Hamburg-Eppendorf. The most important finding is that the 90-day mortality was significantly higher in patients who did not receive gentamicin combination treatment.

Multivariable analyses revealed that gentamicin combination treatment may confer a survival benefit in patients with invasive listeriosis, irrespective of concomitant risk factors. Current recommendations advocate the use of ampicillin and gentamicin for invasive listeriosis. Alternatively, trimethoprim-sulfamethoxazole or meropenem may also be used if ampicillin is contraindicated, e.g. because of medication allergy [4]. The clinical efficacy of gentamicin combination treatment is still subject of debate of controversy. Several retrospective studies have previously failed to show a survival benefit when gentamicin was added to beta-lactam therapy [5]. One study even found that the use of ampicillin in combination with gentamicin was associated with an unfavourable outcome and higher mortality in patients with neurolisteriosis [8]. Notably, our study shows a survival benefit for patients treated with gentamicin combination therapy, despite a higher proportion of patients with neurolisteriosis in the gentamicin combination groups compared with the monotherapy group. However, the largest prospective study to date showed an independent protective effect on survival when gentamicin was used for longer than three days [2]. The results of our study, in which patients in the gentamicin group received gentamicin for at least four days support this benefit of gentamicin combination therapy for invasive listeriosis in another European setting.

Previous studies have shown that neurolisteriosis is associated with a better outcome compared to patients with bacteremia [2]. In our cohort, neurolisteriosis was associated with lower mortality, although not significantly. This could be due to the rather small cohort.

The fact that a considerable proportion of patients with invasive listeriosis did not receive gentamicin combination treatment at a tertiary care center raises concerns about the adequacy of antibiotic therapy in clinical practice. Important potential side effects of gentamicin include ototoxicity and nephrotoxicity. However, several studies have shown a reduced risk of these side effects if serum levels are monitored [9]. Baseline serum creatinine did not differ between the two subgroups, suggesting that it was not acute or chronic renal disease that may have prompted physicians to decide against gentamicin administration due to concerns about nephrotoxic effects. In our cohort, serum levels were monitored regularly, and none of these side effects occurred.

As mentioned above, significantly more patients in the gentamicin combination treatment group had neurolisteriosis than in the monotherapy group. This may suggest that patients diagnosed with neurolisteriosis are more often treated with gentamicin combination therapy than patients with bacteraemia, although the recommended treatment regimen applies to both groups [4]. A plausible explanation for this difference could be that physicians might consider the clinical presentation of patients with neurolisteriosis to be more critical and therefore feel the urge to initiate treatment with gentamicin combination therapy. This hypothesis is supported by the increased frequency of ICU admissions of patients with neurolisteriosis compared with patients with bacteraemia. The older age and higher aaCCI values in patients receiving monotherapy may have influenced the decision not to administer gentamicin to this group as well. However, it is important to note that there were no restrictions or limitations to the therapy in either group.

Rapid diagnosis and clinical awareness are essential for the effective treatment of listeriosis. In our study cohort, the median time from symptom onset to diagnosis was three days in both groups, suggesting that patients in both subgroups presented at a similar stage of the disease.

Our findings of a high aaCCI and a high prevalence of cardiovascular disease, chronic liver disease and diabetes mellitus are consistent with previous studies on risk-factors for invasive listeriosis [3]. As highlighted in the existing literature, steroid use is an important risk factor for invasive listeriosis [3], which is underlined in our patient cohort.

The widespread use of PPIs in our cohort raises questions about possible associations with invasive listeriosis. PPI use increases the risk of bacterial gastroenteritis by disrupting the gastric acid barrier, thereby altering the intestinal microbiota, and reducing the natural defense against exogenous bacteria [10]. A Danish study found that among listeriosis patients aged over 45 years, 23% used PPIs compared with 6% in the control group, suggesting an association between PPI use and increased susceptibility to listeriosis [7]. In Germany, 15% of the population are prescribed PPIs at least once a year [11]. In our study cohort, 80% were taking PPIs at admission, suggesting a possible association with the incidence of listeriosis. While PPI use is often associated with corticosteroid use, which is a risk factor in itself, it is noteworthy that several patients received only PPI without concomitant corticosteroid therapy. Therefore, we recommend a critical evaluation of the indication for PPI treatment.

This retrospective, real-life, single-center study has several limitations. First, the rather small number of cases limits the validity of our results and the ability to correlate numerous variables. For example, we were not able to examine potential differences between ampicillin and meropenem or trimethoprim/sulfamethoxazole treatment on patient outcomes. Second, we would ideally have analyzed neurolisteriosis and bacteraemia as separate entities. We are aware of the limitation of the unequal distribution of these manifestations in the subgroups but were able to mitigate this by including the different manifestations in the multivariable analysis. Third, the duration of gentamicin therapy varied within the gentamicin combination treatment group. However, all patients received gentamicin for at least 4 days, which has been shown to be beneficial in the past [2].

## Conclusion

In conclusion, our study cohort of patients with invasive listeriosis highlights the importance of gentamicin combination therapy to reduce mortality. The fact that a significant proportion of patients in this real-world cohort were treated with monotherapy raises concerns about the adequacy of antibiotic therapy in clinical practice.

## Data Availability

No datasets were generated or analysed during the current study.
